# Area Deprivation Index and Coprescription of Opioids and Sedative Hypnotics

**DOI:** 10.7759/cureus.80422

**Published:** 2025-03-11

**Authors:** Shelby Goicochea, Amal Bhullar, Scott Turner, Benton J Stamper, Christina Guerrier, Raymond Pomm, Kitty Leung

**Affiliations:** 1 Psychiatry, Talkiatry, Jacksonville, USA; 2 Psychiatry, University of Florida College of Medicine, Jacksonville, USA; 3 Clinical Pharmacy, University of Florida College of Medicine, Jacksonville, USA; 4 Epidemiology and Biostatistics, Protean Insights Partner, Jacksonville, USA; 5 Psychiatry, Gateway Community Services, Jacksonville, USA

**Keywords:** adult outpatient clinic, benzodiazepine use, controlled substances, coprescription, hypnotics and sedatives, opioid analgesics, prescription drug, primary care providers, social deprivation index, socioeconomic factors

## Abstract

Background and objectives

The coprescription of an opioid and a sedative-hypnotic (i.e., benzodiazepine or Z-drug) is associated with negative patient outcomes. Although multiple guidelines recommend minimizing this combination, studies demonstrate high coprescribing patterns. The Area Deprivation Index (ADI), a validated surrogate measure of socioeconomic status, was used to assess socioeconomic disadvantage. The objective of this study was to evaluate the current prevalence and the relationship between coprescriptions and ADI scores of patients.

Methods

A single-center retrospective observational study from 2019 to 2022 of adult patients in outpatient clinics with a coprescription (i.e., ≥ 7 days overlap between an opioid prescription and a benzodiazepine or Z-drug prescription) was conducted. A negative binomial model analyzed the percentage change in the incidence of coprescription for every unit increase in national ADI.

Results

A total of 6,846 patients had ≥ 7 days overlap of opioid and sedative-hypnotic prescriptions, comprising a total of 83,560 coprescriptions over the four-year period. A negative binomial regression found a 1.004% increased risk of coprescription for every unit increase in national ADI (p < 0.01), indicating higher socioeconomic disadvantage. The average patient was 57 years old with an ADI of 64.1, a coprescription overlap duration of 23.8 days, and ts (MME) of 47.3. Secondary observations included a significant difference in patient ADI across insurance types (p-value < 0.001). Higher MMEs were observed in white, black, and younger patients.

Conclusions

This study highlights the continued prevalence of coprescriptions and their relationship to social determinants of health. A stronger association between coprescriptions among patients with greater socioeconomic disadvantage (i.e., higher ADI) was observed and underscores the importance of incorporating ADI into prescribing risk assessments.

## Introduction

The opioid epidemic continues to have a significant impact on public health across the United States (US), particularly with the rise of deaths involving synthetic opioids, such as fentanyl [1,2]. In 2021, over 17,000 deaths in the US involved prescription opioids, equating to 45 deaths per day [3]. As many as 83% of heroin users stated that their addiction originated from their misuse of prescription opioids [4].

Benzodiazepines along with non-benzodiazepine hypnotics (commonly known as Z-drugs) have gained attention in recent years as catalysts in this epidemic. Benzodiazepines and Z-drugs act on the Gamma-aminobutyric acid (GABA) receptor through different mechanisms but yield similar results of central nervous and respiratory depression. When taken together with opioids, benzodiazepines and Z-drugs increase the risk of emergency department visits, hospital admissions, and death from drug overdose [5,6,7].

Brandt L et al. in 2022 demonstrated a significantly higher probability of experiencing an overdose event in opioid users who were prescribed a benzodiazepine at baseline [8]. In a study of US Medicare patients who were 65 years or older, those on concurrent prescriptions of opioids and benzodiazepines or Z-drugs were associated with increased out-of-hospital and total mortality [9]. A study done by the Kaiser Permanente system also demonstrated that those exposed to coprescriptions were 20% more likely to have an overdose-related event compared to those exposed to opioids alone [10]. Despite a decrease in overall national opioid dispensing from 2012 to 2020, rates of benzodiazepine prescribing in the outpatient setting nearly doubled from 2003 through 2015 [1,11]. In 2017, more than 30% of prescription opioid overdose deaths involved a benzodiazepine [12]. Additionally, Szmulewicz A et al. found that coprescription of Z-drugs and opioids increased the risk of unintentional overdose by twofold in 30 days and almost threefold in 90 days [13].

Black box warnings as well as CDC Clinical Practice Guidelines for Prescribing Opioids for Pain attempt to address the issue of concurrent use and coprescribing of benzodiazepines and opioids. Despite these efforts, benzodiazepine-opioid coprescriptions continue to increase across the US, which then increase unnecessary healthcare costs and risks for patients [14]. Suvada K et al. in 2022 studied 105,720 combined ambulatory and emergency department patient visits, with an average of 33.2% of these visits resulting in an opioid prescription in patients who had another high-risk medication already prescribed, with higher rates of medication being refilled in the outpatient setting versus new medication prescriptions [15]. Existing literature indicates that much of the coprescription of opioids and benzodiazepines takes place in primary care clinics [16,17].

In a study by Neprash HT at al., longer visits were less likely to include coprescribing of opioids and benzodiazepines [18]. With the growing demand for health care services and the continued shortage of primary care providers, the length of time for patient visits diminishes, making it difficult for evidence-based practices and guidelines to be followed.

The current body of literature related to risk factors for benzodiazepine-opioid coprescribing is limited and varies. In a study by Xu KY at al., patients receiving benzodiazepine-opioid coprescriptions tended to be older males, have a lower income defined as a poverty to income ratio >2, have worsening health, and a higher prevalence of smoking [19]. In a separate retrospective analysis, investigators revealed that opioid users who also filled a prescription for a benzodiazepine were less likely to be men, contradicting the findings from Xu KY at al. [6]. Alternative studies indicate the prevalence of coprescriptions were highest in females, non-Hispanic whites, those over 44 years of age, those with Medicare for insurance, and those on chronic opioid therapy [20].

With escalating healthcare requirements and decreased time and resources allotted to providers, varied efforts have been made to bring awareness of prescription patterns to healthcare providers. Attempts have been tried by institutions in the US to integrate alerts in the electronic medical record when coprescriptions occur; however, it was shown that these alerts had minimal effect on prescribing patterns [21]. In a study by Schaffer AL et al. from 2019, interventions including public subsidy of alprazolam and reducing pack size had minimal benefit for reducing non-evidence-based use [22]. On a more promising note, attempts including mandated prescription drug monitoring programs (PDMP) into healthcare practices did reduce coprescription trends [23].

The discrepancies described above indicate that the dangerous combination of an opioid and a benzodiazepine or Z-drug (sedative-hypnotics) can affect all populations, though there may be populations that are more at risk. The literature suggests that individuals facing socioeconomic challenges may be at a heightened risk. It is also well-documented that health disparities are a major risk factor for cardiometabolic disease. Racial and ethnic minorities and poor populations residing in socioeconomically disadvantaged neighborhoods who have unequal access to necessities including food, healthcare, education, and safety tend to have higher re-hospitalization rates that add extra healthcare costs [24,25].

The Area Deprivation Index (ADI) is a robust, composite measure capturing multiple dimensions of neighborhood-level socioeconomic disadvantage including income, education, housing quality, and employment [26]. Importantly, neighborhood disadvantage has been shown to be independently associated with worsened health outcomes such as higher multimorbidity beyond individual socioeconomic measures [27]. Kurani S et al. utilized ADI to discover that for the most socioeconomically disadvantaged counties the risk of filling an opioid prescription was 72% higher and the risk of drug mortality was 36% higher compared with the least disadvantaged counties in the US between 2012 and 2017 [28]. To our knowledge, no study to date has examined the utilization of concurrent opioids and sedative-hypnotics (i.e., benzodiazepines or Z-drugs) with respect to ADI, an important measure of both individual and environmental factors that impact health outcomes. The objective of the study was to assess if there was an association between coprescription of opioids and sedative-hypnotics in patient populations with higher socioeconomic disadvantages (i.e., higher ADI scores).

## Materials and methods

Data source 

The Institutional Review Board (IRB) approved a retrospective observational study spanning 48 months (January 1, 2019, to December 31, 2022) of prescription data from the electronic health record of a large academic health organization in North Florida. All patient data were de-identified prior to analysis. Given the retrospective nature of the study, which analyzed existing medical records with no direct patient contact and used de-identified data, informed consent was waived. Data were securely stored on password-protected institutional servers in compliance with the Health Insurance Portability and Accountability (HIPAA) regulations and accessible only to IRB-approved study investigators. Adult patients aged ≥ 18 years were included if they had at least one coprescription, defined as a concurrent opioid and sedative-hypnotic (i.e., benzodiazepine or Z-drug) prescription that overlapped by ≥7 days and had at least one outpatient visit from the study site location within the study period. Exclusion criteria included adult patients with an invalid address, a liquid opioid prescription, pregnancy, incarceration, or receiving prescriptions from an outpatient pain clinic. For those included in the study, the following information was collected: patient demographic data (age, sex, race, ethnicity, patient address, and insurance), outpatient clinic address, and prescription data (medication name, dose, prescribed frequency of administration, quantity supplied, refill amount, administration route, prescription start date, indication, and for opioid prescriptions, the milligram morphine equivalents). Prescription dispense quantity, frequency of dosing, refill amount, and start dates were used to determine the duration (in days) of the prescription and the duration (in days) of the overlap between the prescriptions, ensuring those with ≥7 day overlap were included in the study and those with six days or less were excluded. Table 1 lists the opioid, benzodiazepine, and Z-drug medications studied.

**Table 1:** List of prescription opioids, benzodiazepines, and Z-drugs studied.

Drug Class	Prescription Drugs
Opioid	Codeine, Hydrocodone, Oxycodone, Morphine, Fentanyl, Methadone, Tapentadol
Benzodiazepine	Alprazolam, Chlordiazepoxide, Clonazepam, Clorazepate, Diazepam, Estazolam, Lorazepam, Temazepam, Triazolam
Z-drug	Eszopiclone, Zaleplon, Zolpidem

Social determinant of health calculations 

Deidentified patient and clinic addresses were converted to Federal Information Processing Series (FIPS) 12-digit codes, which uniquely identify geographic areas using the US Census Bureau tool and then translated to their national ADI using the latest Neighborhood Atlas data, v3.2. The ADI is a validated measure of neighborhood-level socioeconomic disadvantage reflecting 17 social determinants of health dimensions, including income, employment, education, and living conditions. National ADI scores range from 1-100, with the most disadvantaged neighborhoods represented by a score of 100.

Statistical analysis 

For our primary analysis, a negative binomial regression model was used to estimate the percentage change in the incidence of coprescription of an opioid and a benzodiazepine or Z-drug for every unit increase in patient national ADI.

For secondary analysis, the components of the analysis included in the study were age, gender, ethnicity, insurance status, type of benzodiazepine or Z-drug prescribed, patient ADI, clinic ADI, duration of coprescription overlap, and morphine milligram equivalents (MME). Descriptive summaries included frequencies and percentages for categorical variables and frequencies, means, and standard deviations for continuous variables. Comparisons between low vs. high ADI and insurance types (Medicaid, Medicare, commercial, uninsured, hospital-funded insurance, and other, which included auto insurance) were conducted using Pearson's Chi-Square test. Associations between the predictor variables and the amount of MME prescribed were assessed using a general linear model adjusted for age, patient ADI, clinic ADI, and duration of coprescription overlap as secondary outcomes. The level of significance was set at 0.05. Statistical analyses were conducted using SPSS Version 29 and R.

## Results

**Figure 1:**
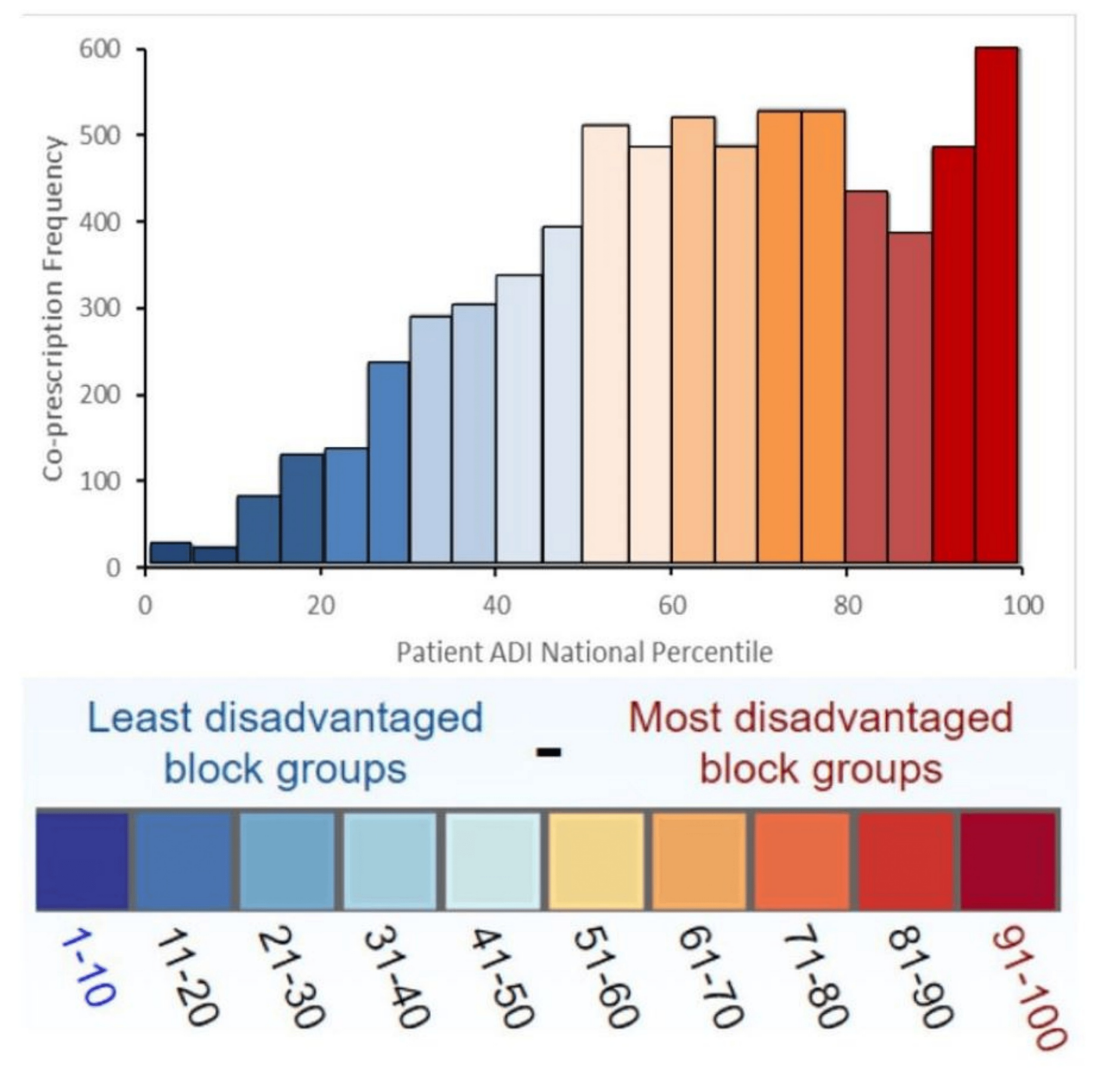
Distribution of patient national ADI across the study period. ADI: Area Deprivation Index.

A total of 6,846 patients were included in the analysis. Descriptive statistics regarding patient demographics were generated and presented in Table 2; the average patient was 57 years old, had a high level of neighborhood disadvantage (average patient ADI of 64.09), a coprescription overlap duration of 23.78 days, was prescribed 47.33 milligrams of morphine equivalents (MME), and was seen in an outpatient clinic with a high level of neighborhood disadvantage (average clinic ADI of 69.03). Notably, the sample distribution of patient national ADI was skewed towards more disadvantaged neighborhoods (i.e., higher patient ADIs) as shown in Figure 1. Pearson's chi-square tests indicated that there was no significant difference in patient ADIs (χ² = 13.616, df = 27, p = 0.9847) across the study time period.

**Table 2:** Descriptive statistics. ^a^ADI: Area Deprivation Index.

	N	Mean	SD
Age	6846	56.98	13.173
Patient ADI^a^	6846	64.09	22.853
Clinic ADI^a^	6845	69.03	24.713
Overlap Duration (Days)	6846	23.78	9.107
Morphine Equivalent	6195	47.33	33.914

Outliers were identified through visual inspection using a box-and-whisker plot of concurrent opioid and benzodiazepine prescriptions. From a clinical perspective, a patient with chronic monthly coprescription of an opioid and a sedative-hypnotic would have a maximum of 48 coprescriptions over a four-year period. Cases exceeding this threshold were considered outliers and removed before re-running the analysis.

To assess the impact of this exclusion, a sensitivity analysis was conducted. The initial negative binomial model, prior to outlier removal, estimated a 1.003% (p < 0.01) increase in the incidence of opioid and sedative-hypnotic coprescription for every unit increase in patient ADI towards a more disadvantaged neighborhood. After removing 328 outliers, the percentage change was 1.004% (p < 0.01) for each unit increase in ADI. Given the minimal difference in these estimates, the exclusion of outliers did not meaningfully alter the results. Instead, their removal helped to mitigate the undue influence of extreme values on the model. Since only 328 outliers were excluded from a total sample of 6,846 patients, their removal is unlikely to have introduced significant bias. However, it is important to acknowledge that excluding high-frequency users may underestimate the true burden of coprescriptions in specific subpopulations. Future research could explore this impact further.

For secondary observations, a general linear model was constructed to analyze the association between the amount of opioid prescribed in morphine milligram equivalents and the predictor variables, which included age, gender, ethnicity, insurance type, type of benzodiazepine or Z-drug prescribed, patient ADI, clinic ADI, and overlap duration. The test of the overall model was statistically significant (F = 10.109, p < .001). Among the predictor variables, age, gender, ethnicity, insurance type, type of benzodiazepine or Z-drug, and clinic ADI were statistically significant at p < 0.001 (Table 3). The adjusted R squared was 0.033, indicating the variance explained by the model. The dosage of opioid prescribed decreased as patients’ age increased. Men were prescribed larger doses of opioids compared to women. For the variable ethnicity, Black and White patients were prescribed larger amounts of opioids compared to other ethnicities, including Hispanics. As the degree of neighborhood disadvantage where the clinic was located increased, the amount of opioid prescribed also increased. None of the types of insurance or benzodiazepines or Z-drugs coprescribed were statistically significant.

**Table 3:** General linear model parameter estimates for secondary outcomes. ^a^ADI: Area Deprivation Index.

Parameter	Beta	Std. Error	t value	Significance
Age	-0.236	0.04	-5.972	<0.001
Male Gender	5.982	0.902	6.631	<0.001
Patient ADI	0.034	0.021	1.588	0.112
Clinic ADI	0.106	0.019	5.557	<0.001
Overlap Duration	0.005	0.046	0.105	0.916
Ethnicity				
Native American	0.485	8.35	0.058	0.954
Asian	6.751	5.023	1.344	0.179
Black	6.113	2.359	2.592	0.01
White	8.378	2.212	3.787	<0.001
Benzodiazepine or Z-drug				
Alprazolam	14.812	13.598	1.089	0.276
Zolpidem	11.007	13.603	0.809	0.418
Lorazepam	10.371	13.636	0.761	0.447
Chlordiazepoxide	9.006	13.607	0.662	0.508
Diazepam	19.897	13.625	1.46	0.144
Eszopiclone	11.201	14.051	0.797	0.425
Temazepam	17.48	13.69	1.277	0.202
Triazolam	20.465	19.223	1.065	0.287
Zaleplon	15.992	15.313	1.044	0.296
Insurance Type				
Hospital Funded	2.774	5.407	0.513	0.68
Commercial	-3.636	2.884	-1.261	0.207
Medicaid	0.978	2.95	0.332	0.74
Medicare	0.709	2.936	0.241	0.809
Uninsured	1.349	3.363	0.401	0.688

The study examined whether differences in insurance type contributed to variation in opioid and sedative-hypnotic prescribing patterns. Prior to analysis, the cohort was restricted to patients with the same insurance type for both the opioid and benzodiazepine or Z-drug visits (n = 6,374) (Table 4). The results of a Pearson’s chi-square test indicated that insurance type varied significantly across the study period (χ² = 588.99, df = 45, p < 0.001). However, when insurance type was included as a predictor in the general linear model analyzing morphine milligram equivalents (MME) prescribed, it was not statistically significant. This suggests that while insurance coverage differed across patients, it did not have a measurable impact on opioid dosages within the cohort.

**Table 4:** Patient national ADI deciles by insurance type. α: Least disadvantaged block group; β: Most disadvantaged block group; *Other insurance including automobile insurance.

		Medicaid	Medicare	Commercial	Uninsured	Hospital Funded	Other*
Patient National ADI Decile	1-10^α^	6	12	24	0	1	0
	11-20	31	58	82	4	0	7
	21-30	48	103	130	6	0	29
	31-40	50	185	246	39	2	13
	41-50	103	262	222	32	3	35
	51-60	173	369	313	56	10	21
	61-70	184	393	318	35	4	7
	71-80	241	373	293	51	9	14
	81-90	254	298	159	33	8	9
	91-100^β^	312	528	133	19	14	10

**Figure 2:**
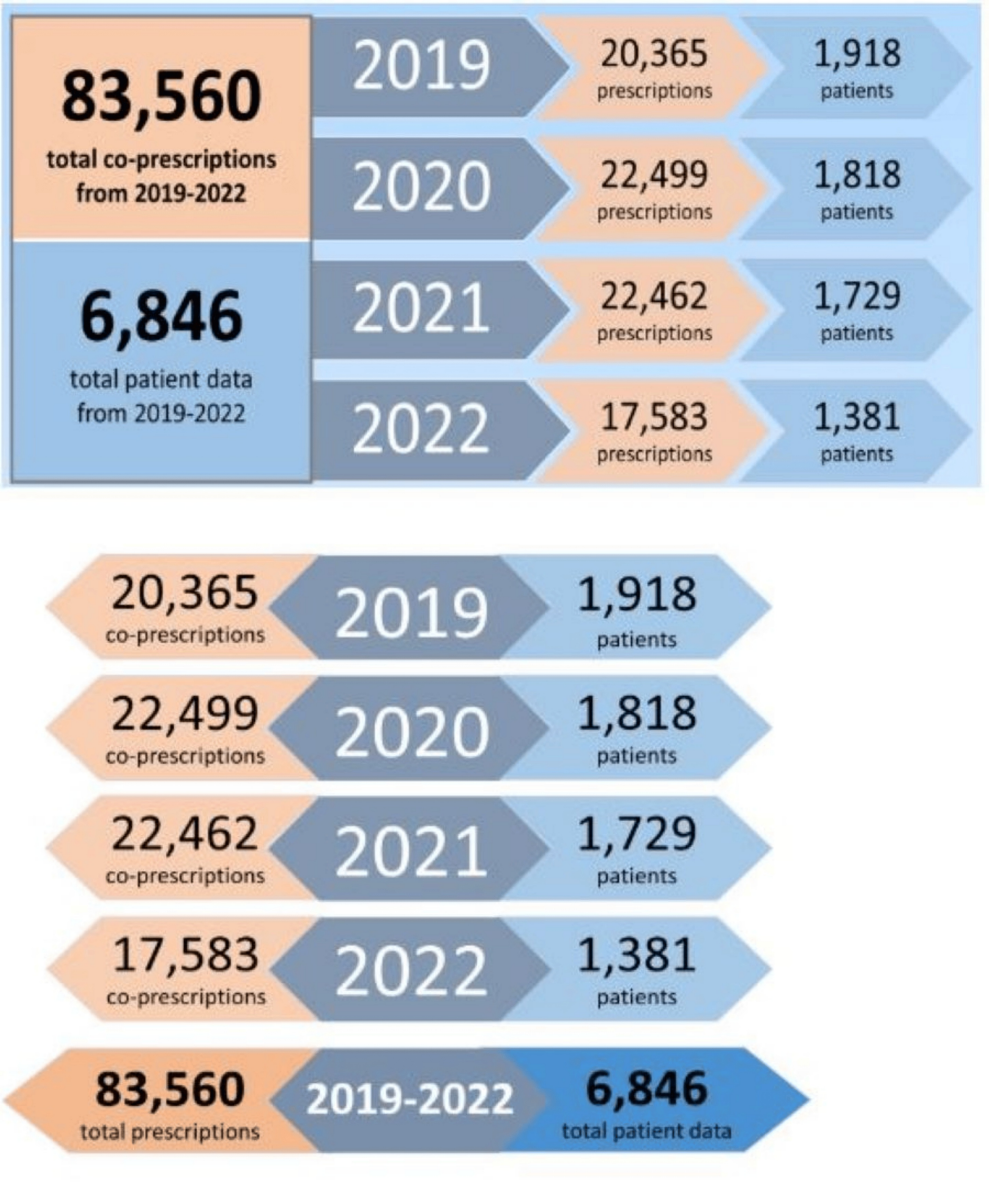
Number of patients and coprescriptions across the study period.

When comparing the number of coprescriptions and patients by year (Figure 2), we observed an increase in coprescriptions from 2019 (20,365 prescriptions) to 2020 (22,499 prescriptions) and 2021 (22,462 prescriptions) and belonging to fewer patients. Coprescriptions decreased to fewer in 2022 (17,583 prescriptions) than they had been in 2019.

## Discussion

Patients living in more socioeconomically disadvantaged areas, as determined by a higher ADI, had a higher prevalence of opioid-benzodiazepine or Z-drug coprescriptions. For every increase in national ADI decile, we identified an approximate 10.04% increase in the risk of having a coprescription of an opioid and a benzodiazepine or Z-drug. Our study results align with previous literature, which indicates that patients with lower income are more likely to use opioids and benzodiazepines or Z-drugs concurrently [17,29].

While the coprescription of an opioid and a benzodiazepine or Z-drug always carries risks, these are not always equivalent [30]. The coprescription overlap criteria were set to greater than or equal to seven days to eliminate short-term, self-limited opioid prescriptions written by Florida prescribers under the “acute pain exception,” which allows for prescriptions of opioids for more than three days up to seven days. This stringent overlap duration was selected to capture patients with more chronic use of coprescription opioids and benzodiazepines or Z-drugs, who would be at a higher risk of adverse events. Within this group of chronic users, elderly patients are particularly vulnerable to the adverse effects of coprescriptions. Our results showed that as patient age increases, the dose of opioid prescribed decreases, suggesting that clinicians are mindful of the increased risks of adverse effects in this patient population.

Coprescriptions continue to be an issue, and efforts should be made to identify vulnerable patients to minimize the risks of adverse events and mortality. Utilizing ADI as a predictor for coprescriptions can potentially help alert clinicians to patients who are at high risk and assist with targeted education or interventions. Additionally, the clinic ADI can serve as a marker to recognize at-risk patients, as we observed that increasing neighborhood disadvantage where the clinic was located was associated with greater amounts of opioids prescribed [31].

When comparing the prevalence of coprescriptions by year, we observed an increase from 2019 to 2020 and 2021, followed by a decline in 2022 to lower levels than in 2019. Further studies should examine the role of the COVID-19 pandemic and its long-term impacts on prescriptions. The pandemic may have increased uncertainty about the future and worsened mental health, leading to increased benzodiazepine and Z-drug prescriptions [32,33].

Whether the higher trend in 2020 and 2021 followed by a decrease was due to COVID-19, telemedicine prescribing permissions, more provider awareness, and education on current guidelines, or other factors could not be determined but is worthy of investigation.

There are several potential reasons why higher ADI is associated with increased coprescription risks. Increased socioeconomic disadvantages encompass factors such as social isolation, limited access to healthcare facilities, limited access to public transit, pollution, unstable housing, and poor health literacy. All these factors pose barriers to alternative healthcare recommendations for diagnoses that opioids and sedative-hypnotics are often prescribed to treat, such as physical therapy, massage therapy, acupuncture, and cognitive therapy for sleep and pain, which may be costly for patients and difficult to access [34].

Our study also found that Black and White patients were prescribed larger amounts of opioids. It has been documented in the literature that racial disparities exist in opioid prescribing, particularly that White patients were more likely to receive opioid analgesics than Black and Hispanic patients [35].

While prior literature suggests that insurance coverage can influence prescribing behavior due to prior authorization requirements, step therapy, or coverage restrictions on controlled substances, the findings of this study do not indicate a strong association between insurance type and opioid prescription amounts. It is possible that other factors, such as prescriber preferences or patient-level characteristics, may exert a greater influence on opioid prescribing patterns. Additionally, socioeconomic disparities, as reflected by patient ADI, may interact with insurance coverage in ways that were not fully captured by this analysis. Future research could examine the role of payer-specific policies in shaping prescribing trends.

Strengths of this study include the use of the ADI as a surrogate measure of socioeconomic disadvantage as a predictor for coprescription of opioids and sedative-hypnotics. ADI has been studied in association with medical conditions, but to our knowledge, no study has investigated ADI with coprescription of opioids and benzodiazepines or Z-drugs. The inclusion of Z-drugs in the analysis is a strength, as recent studies have highlighted similar adverse events compared to concurrent use of benzodiazepines and opioids [13,36,37].

Although there are no current boxed warnings, the inclusion of Z-drugs was clinically significant given their similar risk in combination with opioids. The overlap period selected of seven or more days between an opioid and a benzodiazepine or Z-drug prescription bolstered the analysis to capture patients with chronic use who are most at risk from the adverse effects of coprescription.

Limitations stemmed from the retrospective nature of the study. Causal relationships cannot be inferred from a retrospective study design. Our study only analyzed prescriptions within one healthcare organization but was unable to capture prescriptions from outside the organizations. Although we were able to obtain information on when medications were prescribed from the electronic medical record, when the medication was dispensed from the pharmacy was not obtained. The study's single-center scope may limit the generalizability of the findings to broader populations. Using a retrospective cross-sectional study design, we relied on a snapshot of data at specific time points rather than following individuals longitudinally. The absence of individual identifiers further restricted our ability to track changes in coprescription for each individual over time to assess prescribing pattern changes. This limitation should be considered when interpreting the findings, particularly in relation to prescription practices and their impact on health outcomes over time.

The findings of this study underscore the importance of utilizing ADI as a tool to identify high-risk patients and implement targeted healthcare interventions. Given that neighborhood disadvantage is independently associated with multimorbidity, healthcare systems can leverage ADI to proactively address disparities and reduce adverse outcomes [28]. By integrating ADI into clinical decision-making and public health strategies, healthcare providers can prioritize early screening, preventive care, and tailored interventions for patients in highly disadvantaged neighborhoods [38,39]. From a public health standpoint, ADI could be used to inform resource allocation, ensuring communities with the highest socioeconomic disadvantage receive enhanced access to healthcare [40]. Furthermore, using ADI in population health initiatives can help design policy-driven interventions, such as expanding community-based care models, integrating behavioral health services into primary care, and increasing access to non-pharmacologic treatment options for pain and anxiety to reduce reliance on high-risk medications like opioids and benzodiazepines. Future research should explore the effectiveness of ADI-driven interventions in reducing coprescriptions of opioids and sedative-hypnotics and reducing adverse outcomes.

Future studies can assess patient socioeconomic disadvantage as measured by ADI and its association with adverse events from coprescription of opioids and sedative-hypnotics. Further investigation into predictors of coprescriptions to inform targeted interventions is needed, such as evaluating clinician prescribing trends.

## Conclusions

Overall, our findings suggest a stronger association of coprescribing benzodiazepines or Z-drugs with opioids to patients who have a higher ADI (i.e., more socioeconomic disadvantage). We identified a 1.004% increase in coprescribing risk with every unit increase in national ADI. Unlike previous studies, an increase in coprescribing by year was not identified, although a numerically greater number of coprescriptions was observed in 2020 and 2021. Education for providers to ensure this combination is appropriately dispensed is needed to further limit the potentially lethal effects. Our results, using a novel predictor, ADI, including the 'Z-drugs,' and focusing on coprescription overlap criteria for more chronic use, add to the growing body of literature on the concurrent use of opioids and benzodiazepines or Z-drugs. Future studies investigating patient ADI and its association with adverse drug events from concurrent opioids and benzodiazepines or Z-drugs are warranted.
